# Development of a Multi-Layer Marking Toolkit for Layout-Printing Automation at Construction Sites

**DOI:** 10.3390/s22134822

**Published:** 2022-06-25

**Authors:** Eun Soo Park, Hee Chang Seo, An Yong Lee

**Affiliations:** 1 Department of Architecture, Sahmyook University, Seoul 01795, Korea; espark@syu.ac.kr; 2 BIMFACTORY, Seoul 03081, Korea; heechang.seo@bimfactory.co.kr; 3 Department of Smart Factory, Korea Polytechnics, Incheon 21417, Korea

**Keywords:** layout printing, multi-layer toolkit, marking, construction, robot in construction

## Abstract

In this study, the development of a multi-layer marking toolkit was investigated to improve construction quality and mitigate the problem of irregular designs in the layout-printing work performed at construction sites. The quality of conventional layout-printing work is dependent on the skill of the worker, and construction quality can suffer owing to inconsistencies in drawings resulting from human error. In this study, these problems were analyzed, and a construction-site-layout-marking toolkit apparatus and mechanical unit, with a structure that allowed for multi-layer installation for automated implementation at construction sites, were developed. The marking toolkit and mechanical unit with the multi-layer structure were developed in a modular form so that each module can operate independently. Furthermore, each module was developed in manual mode to improve the system by acquiring information on the movement of the marking toolkit and multi-layer structure. Additionally, data on the layout-printing method was developed by connecting the system via Ethernet and operating a wireless joystick. Finally, experiments were performed on a road surface covered with B4 paper and concrete panels to confirm the operational feasibility of the system, which was developed to operate manually.

## 1. Introduction

Typically, the layout work performed at construction sites corresponds to floor-marking work that denotes the places on the floor where foundations, columns, and walls will be erected and is achieved using chalk boxes, chalk, string, and chalk lines.

Marking work refers to the process of indicating wall thicknesses, including finishing materials, as well as the locations of windows and doors in detail by layout printing major points, such as the shape or dimensions of the floor of the facility and the location of finished surfaces based on the final confirmed and detailed construction drawings after on-site inspections have been performed [1].

In the conventional marking process, the user pulls a chalk line from a chalk box and uses a hook to fix the chalk line to one end of the target object during construction work. The user then plucks the chalk line to mark the target object with a line of chalk [2].

**Figure 1:**
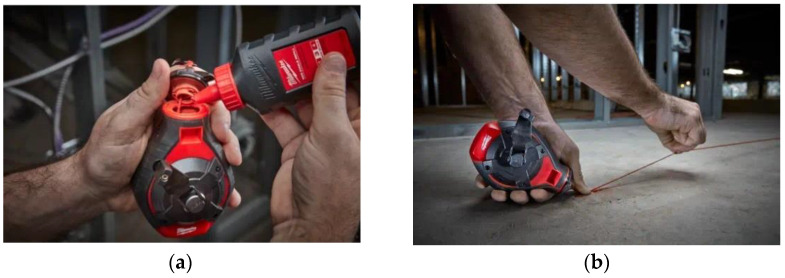
Conventional manual layout-printing work process example: (**a**) filling with powder and (**b**) snapping a chalk line.

The user then uses the line of chalk to cut or attach objects to the target object. Even now, when construction work is performed, as shown in Figure 1, a separate measuring device that can measure length, such as a measuring tape, is used to measure length. When marking work is performed, a significant amount of manpower is required to hold the chalk line at both ends and check the drawings [3].

Furthermore, this method is dependent on the worker’s technical skills and is suitable only for small-scale work [4]. Additionally, it poses problems due to the possibility of a large number of errors, and an increased amount of time is required for accurate measurements to be made [5]. At sites where the construction quality is not uniformly designed, inconsistencies can occur between the site and construction drawings [6].

Looking at the related works, Jensfeld et al. (2006) conducted an experiment to increase the accuracy of marking to within a stable range through laser sensors. As a result of the experiment, accuracy could be improved through spatial modeling as a display point on a laser scanner. Inoue et al. (2011) developed a high-precision positioning system using a survey system. Cao et al. (2021) established spatial location coding rules through spatial location information identification, transmission, and big data analysis, and conducted engineering experiments for spatial coding. Tsuruta et al. (2019) conducted a study to improve the designated position display based on the deviation between the bottom display position and the robot.

Overall, the direction of the research in this area has developed as the technology for location accuracy in layout printing has developed. This study focused on improving the basic marking method in consideration of the actual environment. This study used a multilayer concept to improve the construction efficiency and considered layout-printing work with two or more lines at the same time. The multilayer technology of this study enabled the addition of a line’s color and simultaneous printing of two lines. In addition, this technology can be effectively applied not only to layout printing but also to improve the working environment (cleaning module) and quality checking (vision module) compared with the existing technology.

In this study, we aimed to resolve the problems of conventional marking techniques that are manually performed so that marking work can be efficiently performed in a variety of construction environments using a marking toolkit. Furthermore, in this study, we aimed to provide a construction-site-layout-marking toolkit apparatus that allows for the installation of marking toolkit items on multiple layers.

In Section 2, the methods and current problems associated with on-site marking are described. In Section 3, attachable/detachable marking toolkit modules and a multi-layer structure are proposed. In Section 4, a development system is discussed for the marking toolkit modules. Finally, in Section 5, the results of simple experiments performed on road surfaces are presented. The main scope of this study was as follows:

- Analyze the characteristics of marking applications;
- Design marking modules that consider the properties of construction sites;
- Develop a marking toolkit that can be changed according to the work environment;
- Create a multi-layer structure that considers work efficiency and marking scope;
- Integrate a sensor-linking system to allow for robot operation and incorporate site characteristics.

## 2. Layout Printing’s Application Scope

**Figure 2:**
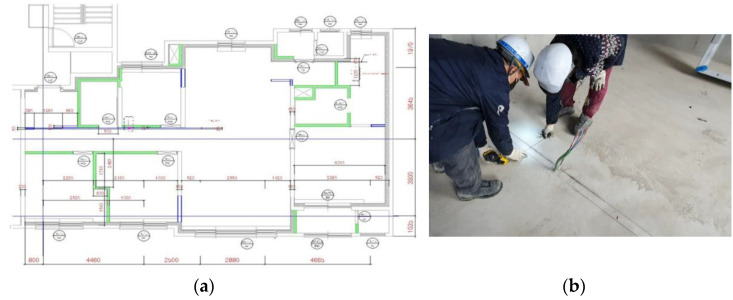
Layout printing: (**a**) layout printing of a floor plan; (**b**) photo of actual work.

In conventional, traditional layout-printing technology, the precision of reference line work may vary based on the skills of the on-site workers, such as carpenters, as shown in Figure 2, and design/construction errors and problems with their associated reduced construction quality can occur due to the fallible know-how and skill of workers at actual construction sites [7].

To minimize the precision required in the work performed by skilled workers, measurements are typically performed at construction sites using equipment such as laser levels and MEP (mechanical electronic plumbing) layouts [8]. Laser levels, which are typically used to measure and mark locations, are used to confirm the vertical and horizontal reference lines for marking work and to check for errors [9].

MEP layouts are devices that can check locations in CAD (computer-aided design) drawings and BIM (building information modeling) data in real time at construction sites to ensure that work can be performed. Generally, they are used for layout printing, which requires precision and are not very useful for marking alone.

**Figure 3:**
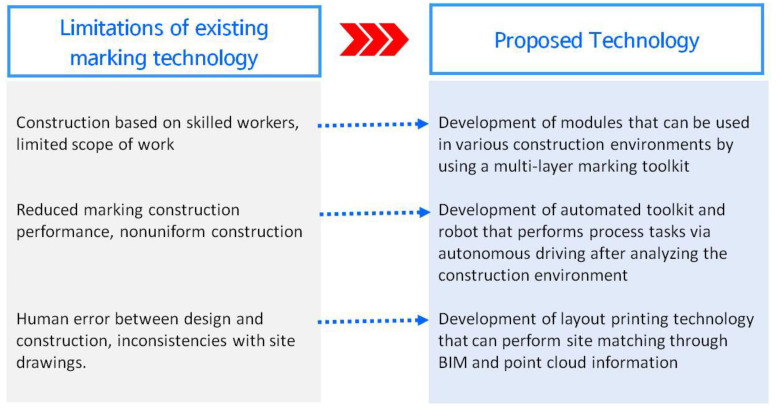
Scope of layout-printing technology that considers process characteristics.

Owing to the recent expansion of the BIM construction market and the increase in irregular construction sites, there is a need for improvements in the drawing technology of construction sites that links design and construction [10]. As shown in Figure 3, the efficiency of the existing marking technology at a construction site must be improved and construction quality must be ensured.

- It is necessary to mitigate the problem of human error that occurs between the design and construction during the marking work.
- Improving construction accuracy is necessary because of the increase in construction sites with irregular geometrical shapes.
- There is a need for 3D spatial information sharing and construction site inspection technology owing to BIM design.

This study is part of the development of a multi-layer apparatus and toolkit in modular form for developing mobile self-driving robots. To overcome the aforementioned technical limitations of conventional marking, in this study, we attempted to make improvements by combining different technologies, which allows for the selection of marking toolkit items based on the road surface and technology that enable multi-layer layouts [11]. The aim of this study was to overcome the work scope limitations of construction, which is dependent on skilled workers performing conventional marking work, and to develop marking technology for a variety of functional improvements.

## 3. Mechanical Design

Self-driving robots are now being used in a large number of commercial applications. Unlike industrial robots that perform simple and repetitive tasks, they share the same environment as humans and are being developed into human-friendly intelligent service robots. In contrast to determining efficient marking methods for paint and rubber based on road conditions, in this study, we developed a marking tool that can be used to efficiently improve marking quality by switching (attaching and detaching) marking tools based on the conditions of the site.

### 3.1. Marking Toolkit Modules

If only one tool is used to perform work at a construction site, then it is difficult to adequately respond to changes in the state of the site’s floor surface, and the floor surface can be damaged or the marking quality can suffer [12]. The toolkit presented in this study was developed in a form that can be mounted onto a slide structure so that tools can be quickly switched and installed according to the state of the construction site.

**Figure 4:**
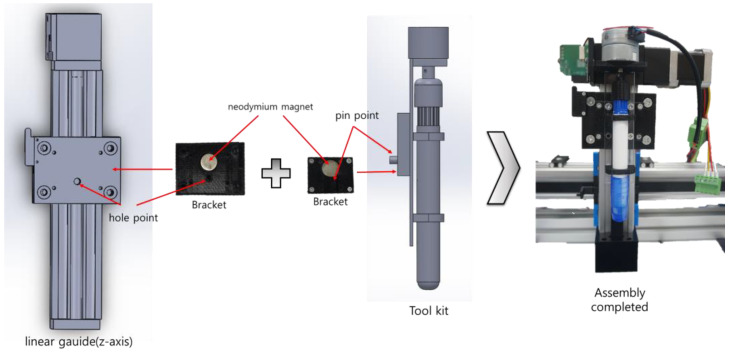
Structural diagram of the attachable/detachable toolkit.

To develop marking tools with a form similar to chalk-type tools to match road surface conditions, in this study, we developed a pen-type marking toolkit with a form that allows for adjustment of the length of the pen tip by rotating it, as shown in Figure 4.

The main goal of this study was to develop an automated robot that replaces existing layout-printing work. This study first selected the pen type, the most generalized form, as an early model of marking automation. In this process, the printing technology of the marking robot was intended to utilize the most basic marking technology. Pen-type technology has an advantage in terms of marking accuracy because there is no separation distance. In addition, since the layout-printing environment is sensitive to external factors, such as curvature and drivability of the road surface, the printing element technology was simplified with a prototype to increase the accuracy. The current pen type simultaneously involves considering the length adjustment according to the rotation of the pen tip and the impact of the wheel. In the future, after stabilizing the driving technologies in terms of drivability and location recognition, spray and laser marking types will be additionally devised.

A step motor (gear: 43:1) was attached so that the length of the pen tip increased or decreased by approximately 2 mm per rotation until it reached the desired length. Furthermore, as shown in Figure 4, to facilitate attachment and detachment, pins and holes were added to the attaching tool parts to reduce the attachment position errors, and neodymium magnets were used to increase the efficiency of the attachment during bolt assembly.

**Figure 5:**
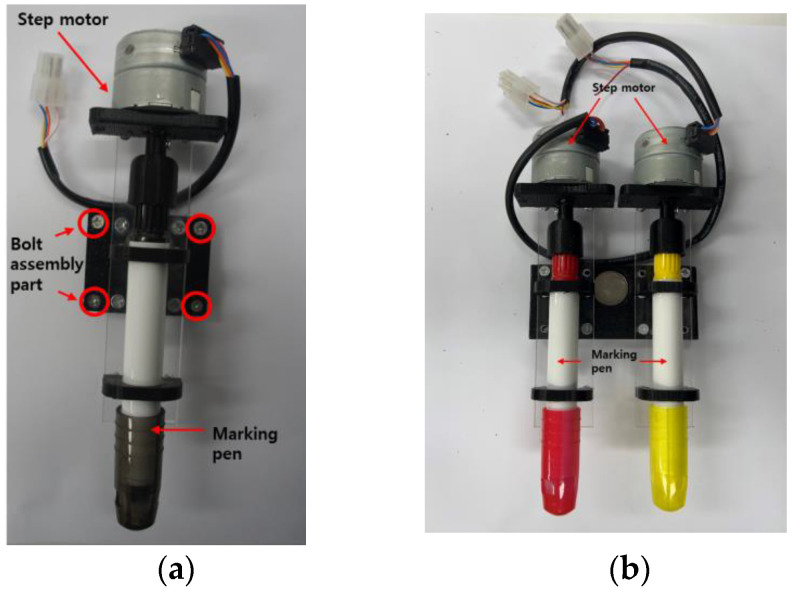
Marking toolkit modules: (**a**) single-type; (**b**) double-type.

A pen-type toolkit module was attached to a driver, and the driver that operates the toolkit was configured as a connector. Hence, it is easy to attach and detach modules. Additionally, two pen-type modules were manufactured so that they could be selected based on the repetitive layout work on the road surface and the layout width and color. Each pen can move independently, and they were designed to be detachable. Various toolkit items were developed to enable their selection according to the road surface. As shown in Figure 5, a pen-form toolkit (single pen, double pen) was developed to efficiently perform marking. The toolkits were developed with a structure that is the same as that of the brackets and can be attached and detached efficiently.

**Figure 6:**
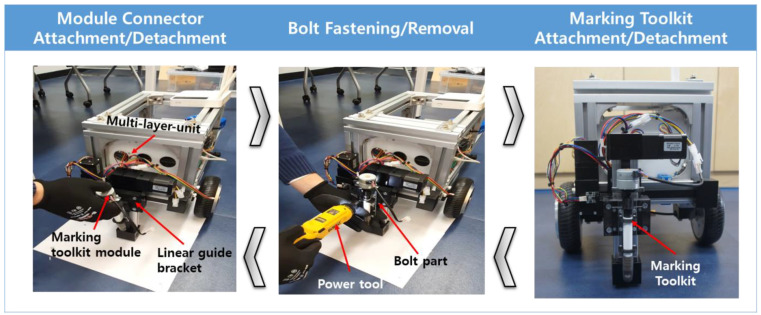
Marking toolkit attachment and detachment test process.

The detachment and attachment lead times of the toolkit were measured four times to test the detachment function of the developed marking toolkit brackets, as shown in Figure 6. In the detachment tests, it was possible to perform detachment at an accurate position due to the developed bracket magnets; it was also possible to perform assembly and disassembly quickly and easily within an average of 35.1 s by just assembling the bolts and connecting the connectors to fasten the toolkit items to the mechanical unit.

The order of detachment and attachment is as follows:

1. To attach the marking toolkit, temporarily assemble the marking toolkit to the bracket and the linear gate bracket.
2. Using an electric tool, attach the marking toolkit in the assembled state to the position of the bolt shown in Figure 5.
3. Assembly of marking toolkit is completed.

The detachment order proceeds in the reverse order of the attachment order so that it can be easily detached.

**Figure 7:**
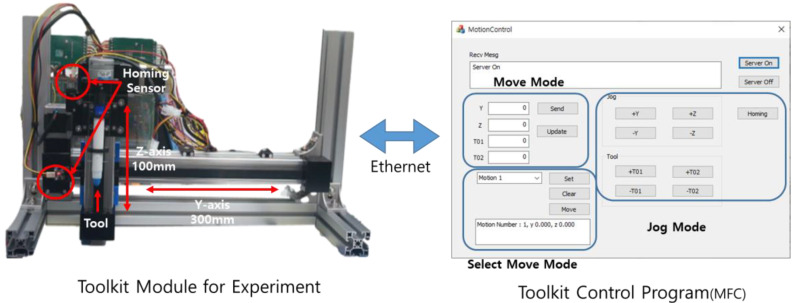
Toolkit Module for the Experimental System.

To test the attached toolkit with a drive shaft, a mechanical unit test device, which can move along the y- and z-axes, was created. Two-step motors were attached along each axis via stm32 so that the device can move to a user-specified position along the z- and y-axes. A homing sensor was attached along each axis to ensure that the device can move to a reference point and then move to a user-specified position. Furthermore, the device was developed so that the user can independently operate one or two specified tools in the attached toolkit. An Ethernet-based tool that can perform regular tests was created to test the tool, as shown in Figure 7.

In Figure 7, the system for the regular test was configured to allow for the removal of the drive shaft and toolkit through the PC control program. The system can be individually removed from each axis via Ethernet communication. It was designed to be able to operate in the movement and jog modes, which are functions of the control box. It also supports a selected move mode that can be used at the desired time by setting the coordinates of the desired location.

### 3.2. Multi-Layer Mechanical Unit

**Figure 8:**
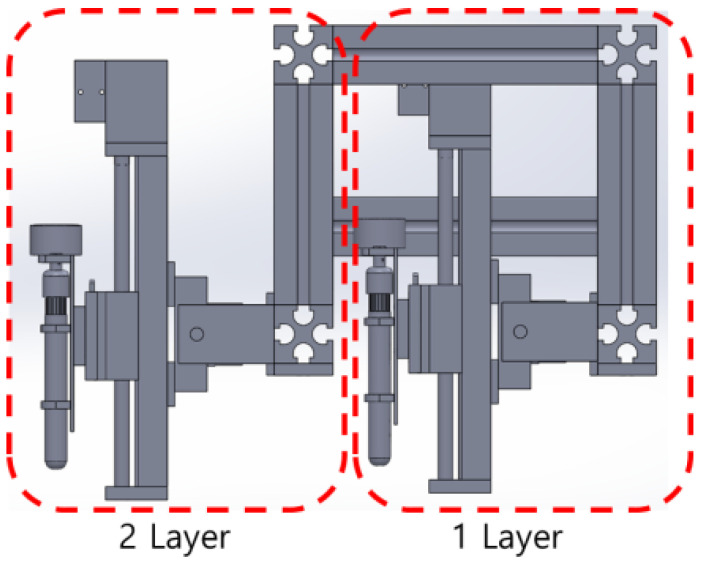
Multi-layer mechanical unit with a vertically arranged structure (side view).

A multi-layer structure was developed, as shown in Figure 8. Hence, it is possible to selectively attach marking toolkit items and install multiple toolkit items. This, in turn, allows for the application of the layout’s width, line shapes, and line colors onto the road surface. The toolkit items were arranged vertically so that layer 1 and layer 2 can operate independently. Therefore, it is possible to efficiently draw the layout, including the road surface conditions, and the size and shape of the lines. The structure was developed to ensure that single- or double-toolkit items with different shapes can be attached. Hence, it is possible to mix toolkit items as required.

**Figure 9:**
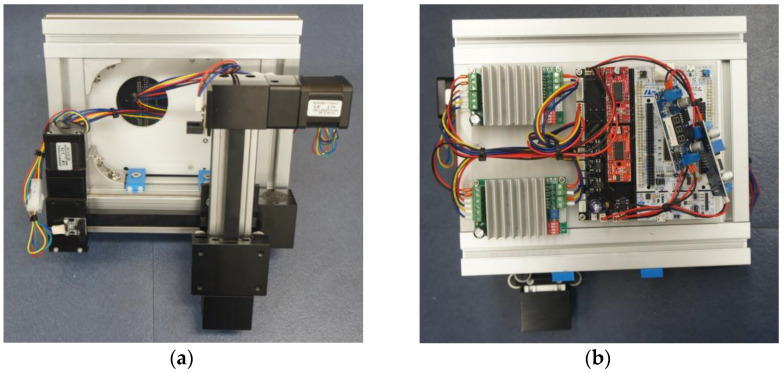
Multi-layer mechanical unit with a modular form: (**a**) front view of the multi-layer mechanical unit and (**b**) plane view of the multi-layer mechanical unit.

Additionally, as shown in Figure 9, the front was constructed with the drivers to ensure that the marking toolkit can be operated. The back had four stm32-based step motor drivers attached to it, and it was constructed in a modular form that allows drivers and toolkit items to operate. A single-module form was developed, as this allowed for easy attachment and detachment. Single and double toolkits were developed with structures that the user can attach according to the conditions of the road surface or lines.

Moreover, given that the mechanical unit is modular, it is easy for the user to respond in the event of a malfunction. Therefore, the mechanical unit was developed in an integrated manner so that it is efficient in terms of maintenance.

**Figure 10:**
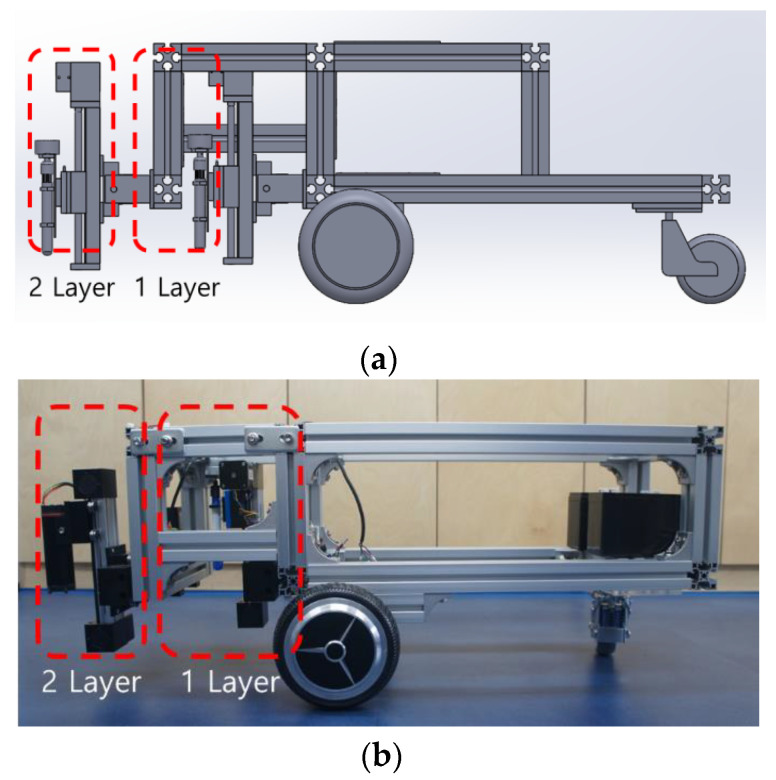
Profile of the multi-layer mechanical unit and mobile robot: (**a**) multi-layer mechanical unit design diagram and (**b**) multi-layer mobile robot.

A multilayer mechanical unit was developed that can be attached to a mobile robot with two wheels and one caster, as shown in Figure 10. The multi-layer mechanical unit can perform printing as it moves along the y- and z-axes, and the mobile robot can move along the x-axis. Thus, it is possible to print on the road surface according to the required line shapes.

Therefore, in this study, the marking toolkit was interlocked with the multi-layer mechanical unit, and layout printing was performed through the multi-layer machine unit according to the movement of the mobile robot. The functional role of the marking toolkit was defined as follows:

1. When printing two or more lines, it is possible to operate freely while moving according to the printing position of two lines.
2. It can be used to accurately display the linear section while driving the robot.

## 4. Marking Functionality Experiments

### 4.1. Experimental Layout

Two types of experiments were conducted using the developed marking toolkit. For the road surface condition, the experiments were conducted with sheets of B4 paper attached to the floor surface, as well as concrete floor panels that could be marked. Among the multiple layers, where the marking toolkit could be attached, the toolkit was attached to the mobile robot in layer 1, and printing was performed as the multi-layer mechanical unit was moved 800 mm along the y-axis.

### 4.2. Basic Test for Floor Surface Marking Functionality 

**Figure 11:**
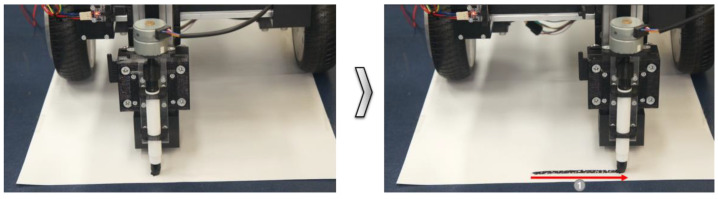
Before and after the y-axis movement marking functionality test.

As shown in Figure 11, the toolkit drive operated normally in direction 1. It was confirmed that printing occurred on the B4 paper that was attached to the floor surface. The marking thickness and precision were affected by the separation distance from the floor surface. Additional experiments were conducted to perform precise marking by considering the separation distance from the floor surface.

**Figure 12:**
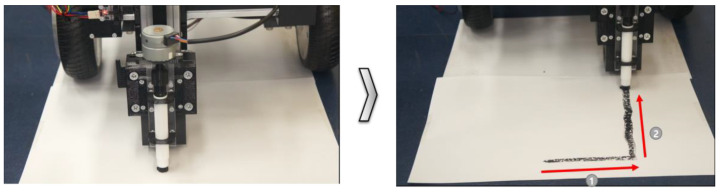
Before and after the x-axis movement marking functionality test.

In additional experiments, marking tests were performed by moving the mobile robot with a toolkit attached at 900 mm along the x-axis. The experimental results showed that the mobile robot operated normally when the movement direction corresponded to direction 2, as shown in Figure 12. The results of repeated experiments showed that functional improvements were required to improve the accuracy of the lines when the mobile robot moved. After the tests on the paper floor surface, concrete test panels were designed to accurately test the floor in a construction environment. 

### 4.3. Concrete Floor Surface Marking Functionality Test

**Figure 13:**
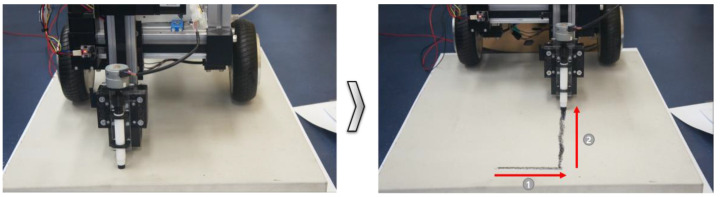
Before and after the concrete floor surface marking functionality test.

For the concrete panels, a simple layout-printing functionality testbed was created by requesting the production of concrete panels to create a marking work environment during the construction of a testbed for similar construction site experiments. In the tests on the concrete panels, the same marking type was used (a single-pen type) to confirm whether marking was possible. The results of the repeated tests showed that the toolkit operated normally on the concrete floor in the functional tests, and the line markings were accurate in the marking tests, as shown in Figure 13.

## 5. Discussion: System Component Modules and Integration Process for Layout Printing

**Figure 14:**
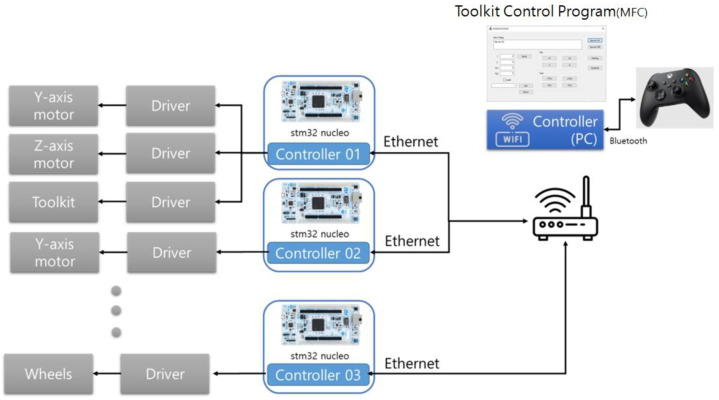
Overall system diagram.

To operate the toolkit, multi-layer mechanical unit, and mobile robot, in this study, an MCU stm32 (32-bit microcontroller) and a FreeRTOS (free real-time operating system) were installed on each driver to ensure operation with a real-time clock, as shown in Figure 14. The system was developed so that each function (including the home sensor, motor control, and Ethernet communications) can operate periodically in real time. For the developed modules, a system capable of proactive learning was developed. Hence, the user can suggest methods via manual operation as opposed to automatic operation to analyze the operational functions and work functions that are required for layouts.

A Bluetooth joystick was connected to enable the manual operation to easily conduct the layout-printing experiments. This joystick was linked to a Windows-based user program. Hence, it is possible to move the toolkit attached to the multi-layer mechanical unit and operate the mobile robot.

**Figure 15:**
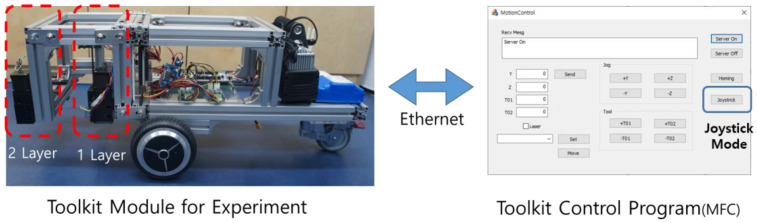
Multi-layout Marking Toolkit Robot for the Experimental System.

As shown in Figure 15, the server was constructed based on the MFC (Microsoft Foundation Class) and each driver’s controller can access it as a client and operate it via the joystick. To check the status of the layout printing, according to the road surface conditions, the system was developed so that work can be performed in an environment that is similar to manual work using a joystick.

In addition to the spatial information modeling work at the site, the work equipment that can be considered during the research process can be classified into two categories: the sensor and control category and the removal equipment category; Table 1 lists the components of the main modules for each R&D system.

**Figure 16:**
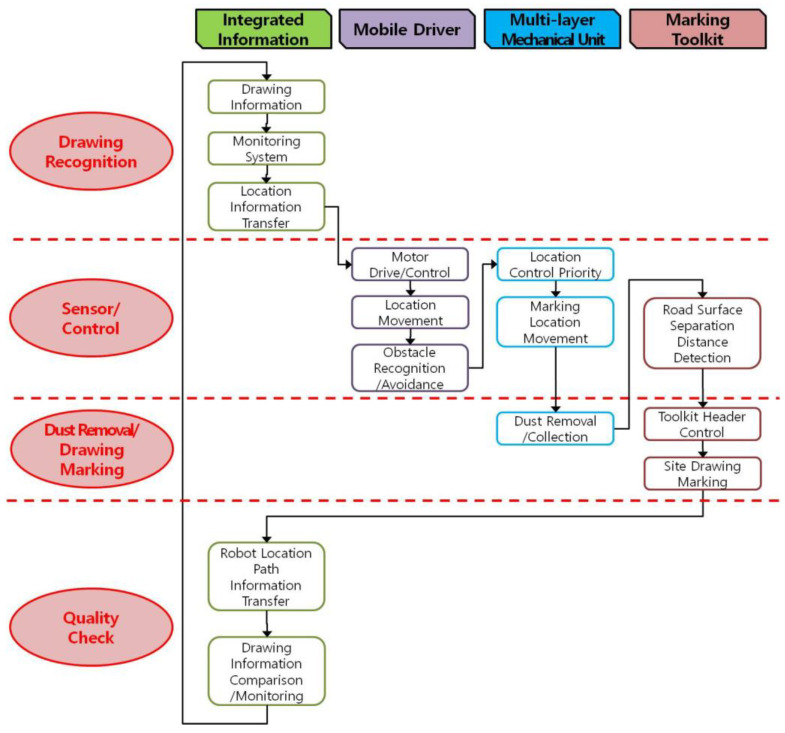
Integrated, automated layout-printing robot concept and process.

Figure 16 shows the basic process for an integrated, automated construction-site-layout-printing robot system concept that will be improved in the future based on this study.

As shown in the figure, the layout-printing process consists of four parts, as shown along the top row. Through Figure 16, the direction of this study represented the overall work process and the application of the currently developed multilayer marking toolkit technology. Among the four parts, the multilayer mechanical unit and marking toolkit module were intensively reviewed in this study. The multilayer mechanical unit corresponds to spatial location technologies that were intensively investigated in several previous studies. This part needs a very important technical mechanism. This study focused on dust removal and drawing marking rather than the sensor and control part. In particular, the multilayer marking toolkit can expand its utilization, allowing for additional functions such as sensor modules, removal modules, marking modules, and quality check modules to be deployed. This can be a good means to improve the construction performance in a new attempt to automate layout printing.

## 6. Conclusions

To resolve the problems of conventional marking technology, in this study, we proposed a marking toolkit and construction-site-layout-marking toolkit apparatus that is capable of multi-layer installation so that the marking toolkit can be used efficiently in a variety of construction environments. To improve the marking process and mitigate problems with environmental conditions, in this study, we developed attachable and detachable marking toolkit modules and a multi-layer structure. Furthermore, to verify the operation of the marking toolkit, a system was constructed and experiments were performed on a floor surface covered with B4 paper and concrete panels.

Based on the experimental results, the functional marking elements were able to obtain satisfactory layout-printing results according to the drive direction. However, in the case of the pen type, problems could potentially occur in which the marking thickness and accuracy were not consistent due to the roughness of the construction site floor. In the layout-printing process, the core function of this technology involves providing marking accuracy by moving the positions of the mobile drivers and marking toolkit parts.

In the conventional layout-printing process, specifying marking points and snapping chalk lines are important processes that require skill. To implement real-world robots, it is very important to enhance the technology for improving marking precision. Hence, it will be necessary to adopt the following development approaches to improve functionality in the future.

(1) Test and verify automated drive functions in stages:

Acquire data on the mobile robot-based multi-layer structure and marking toolkit via a variety of approaches in manual mode, where the user directly operates the robot. The functions are then added and verified in stages so that the robot can operate in an automated mode.

(2) Improve the printing accuracy based on data that considers the site environment:

Promote follow-up research that builds a database to enable layout printing in various environments via deep learning based on operational data.

## Figures and Tables

**Table 1:** Main system modules for layout printing.

Category	Module	Composition
Integration information	Monitoring module	Drawing information Robot location path information Path comparison analysis
Sensor/control	Object recognition sensor module	Vision sensor system Artificial intelligence object recognition/avoidance function
	Location movement control power module	Location control equipment Drive module
	Road surface separation distance sensor module	Sensor distance measurement Toolkit header control
Drawing markings	Marking module	Marking-type (pen, laser, etc.) module Marking toolkit
	Dust removal module	Debris suction module Debris storage Wet/dry vacuum system
